# Chemical Profiling and Bioactivity of Microalgae Extracts for Enhancing Growth and Anthracnose Resistance in the Common Bean (*Phaseolus vulgaris* L.)

**DOI:** 10.3390/biotech14010017

**Published:** 2025-03-08

**Authors:** Alessandro A. dos Santos, Camila Nader, Mateus B. de Freitas, César F. Ribeiro, Geovanna de Oliveira Costa, Louis P. Sandjo, Alex S. Poltronieri, Roberto B. Derner, Marciel J. Stadnik

**Affiliations:** 1 Laboratory of Plant Pathology, Agricultural Science Center (UFSC-CCA), Federal University of Santa Catarina, Florianopolis 88040-900, SC, Brazil; mateus.brusco@ufsc.br (M.B.d.F.); cesarfreitasr@gmail.com (C.F.R.); marciel.stadnik@ufsc.br (M.J.S.); 2 Laboratory of Algae Cultivation, Agricultural Science Center (UFSC-CCA), Federal University of Santa Catarina, Florianopolis 88040-900, SC, Brazil; kamila_nader@hotmail.com (C.N.); roberto.derner@ufsc.br (R.B.D.); 3 Laboratory Natural Products Chemistry, Physical and Mathematical Sciences Center (UFSC-CFM), Federal University of Santa Catarina, Florianopolis 88040-900, SC, Brazil; cogeovanna@gmail.com (G.d.O.C.); p.l.sandjo@ufsc.br (L.P.S.); 4 Laboratory of Entomology, Agricultural Science Center (UFSC-CCA), Federal University of Santa Catarina, Florianopolis 88040-900, SC, Brazil; alexpoltronieri@gmail.com

**Keywords:** biostimulant, hydroalcoholic extract, microalgae

## Abstract

The present study aimed to chemically profile the hydroalcoholic extracts from the microalgae (MEs) *Nannochloropsis oculata*, *Phaeodactylum tricornutum*, *Tetradesmus obliquus*, and *Tetraselmis tetrathele* and evaluate their effects on the development of *Colletotrichum lindemuthianum* and anthracnose symptoms, as well as on the initial growth of bean plants. For this, MEs were analyzed using UPLC coupled with a mass spectrometer, allowing the identification of peaks and annotation of potential metabolites. Fungal mycelial growth was assessed seven days after inoculation, and conidial germination was measured 72 h after incubation, using ME concentrations of 0, 0.1, 0.5, and 1.0 mg·mL^−1^. Bean seeds of the IPR Uirapuru cultivar were sown and treated with 3 mL of extracts at four time points: at sowing and 72 h after each previous treatment. After 11 days of cultivation in a growth chamber, the plants were divided into two groups: one for anthracnose control assessment and the other for evaluating growth promotion by MEs. Plant length as well as fresh and dry weights of shoots and roots were determined, leaf pigments were quantified, and anthracnose severity was assessed using a diagrammatic scale. The UPLC analysis identified 32 compounds in the extracts of the four microalgae, belonging to different chemical and functional groups, with lipids being the most significant fraction. The extracts exhibited variability and diversity in chemical composition depending on the microalgal species. MEs did not affect mycelial growth yet increased the germination of *C. lindemuthianum* conidia, regardless of the dose or species used. Anthracnose severity was not affected by the microalgae extracts. Regarding growth promotion, the extracts showed varying effects but generally increased shoot and root length, fresh biomass, and leaf pigment content.

## 1. Introduction

Beans (*Phaseolus vulgaris* L.) are a staple food in the global diet and play a crucial role in smallholder farming, particularly in South America and Africa [1,2]. However, the productivity of this crop has been often threatened by both abiotic and biotic adverse factors. Among the last ones, the fungal disease anthracnose (*Colletotrichum lindemuthianum*) has been considered one of the most important factors being particularly difficult to control due to diversity of races and efficient dissemination of pathogen. Under optimal humidity and temperature conditions, it can cause production losses of up to 100% [3,4,5].

The intensive use of pesticides has raised concerns regarding the presence of toxic residues in the environment and potential risks to human health. In this context, microalgae have emerged as a sustainable alternative to synthetic agrochemicals, attracting increasing interest due to their broad potential applications in agriculture [6,7]. Moreover, they can be utilized both as biostimulants for plant growth and as biological control agents against pathogens [7,8].

Microalgae exhibit a wide range of bioactive compounds that can function as biostimulants and resistance inducers in plants. These compounds promote biochemical adjustments and physiological modifications, improving root system architecture and nutrient cycling [9]. Additionally, microalgae produce various secondary metabolites with antibacterial and antifungal properties, capable of inhibiting the activity of phytopathogens such as fungi, bacteria, and nematodes [10,11].

Among the most extensively studied genera, *Chlorella* and *Arthrospira* stand out due to their bioactive compounds, including polysaccharides, proteins, and lipids, which exhibit plant growth-promoting, antimicrobial, and antioxidant properties [6,8]. Studies have demonstrated that *Arthrospira platensis* enhances growth and biomass accumulation in various crops, such as maize (*Zea mays* L.), tomato (*Solanum lycopersicum*) [12,13], and pepper (*Capsicum annuum*) [13]. On the other hand, extracts of *Chlorella vulgaris* have shown antifungal activity against various phytopathogens, including *Aspergillus niger, Cercospora beticola*, and *Fusarium oxysporum* f. sp. *melonis* [6,14,15].

These findings underscore the promising role of microalgal compounds in promoting sustainable agriculture by reducing the use of chemical inputs and enhancing crop resilience. However, studies focusing on microalgae from the genera *Nannochloropsis*, *Phaeodactylum*, *Tetradesmus*, and *Tetraselmis* still remain limited concerning plant protection and growth promotion. Given this scenario, the present study aimed to characterize the compounds present in hydroalcoholic extracts of four microalgal species, evaluate their effects on common bean plant growth, and investigate their efficacy in controlling *C. lindemuthianum*.

## 2. Materials and Methods

### 2.1. Biological Material

The microalgae used in this study (Table 1) were maintained and cultivated in the Algae Cultivation Laboratory (LCA) of UFSC. For that, the experimental cultures of *T. obliquus* were developed in 2L borosilicate Schott flasks containing LCA-AD medium [16], while *P. tricornutum*, *T. tetrathele* and *N. oculata* were developed in LCA-AM medium [17]. The cultures were maintained at a temperature of 24 °C, with constant agitation by bubbling atmospheric air enriched with 0.2% CO_2_ (*v/v*), irradiance of 200 µmol photons·m^−2^s^−1^, and in a continuous photoperiod. After reaching the stationary phase, cultures were collected centrifuged (NT825, NovaTecnica, Piracicaba, Brazil) at 3500 RPM, −4 °C for 5 min, lyophilized (70040, Labconco, Kansas City, MO, USA), and stored at 5 °C until use.

The isolate MANE 003 of race 73 of *C. lindemuthianum* was used in the experiments. The fungus was maintained on PDA medium, and the spores were obtained after 15 days of fungal growth on bean pods as described by De Freitas and Stadnik [4]. The conidial suspension was obtained by adding sterilized distilled water to the tubes. The tubes were vigorously agitated to dislodge the conidia, and the resulting suspension was filtered through gauze to remove mycelial fragments. The filtrate was then subjected to three successive centrifugation steps (5810R, Eppendorf, Hamburg, Germany) at 10,000 RPM and 25 °C for 10 min. Finally, the conidial concentration was measured using a Neubauer counting chamber and then diluted as required for each experiment.

The bean cultivar IPR88 Uirapuru (Agronomic Institute of Parana, IAPAR, Curitiba, Brazil), which is susceptible to race 73 of *C. lindemuthianum* [4], was used in the experiments. Bean seeds were sown in plastic conic tubes (75 cm^3^) filled with vermiculite of medium particle size, pH 6 to 9, and maximum humidity (*w*/*w*) of 8% and incubated in a growth chamber for 11 days. The chamber conditions were maintained at 22.5 °C, 80% relative humidity, and a 12 h photoperiod. The substrate was watered daily (except when treatments were applied) with 3 mL of sterile distilled water per tube.

### 2.2. Obtaining of Hydroalcoholic Extracts

The microalgae extracts (MEs) were obtained as previously described [18], with modifications. For that, the lyophilized biomasses were agitated in ethanol 70% (35 mg·mL^−1^) at 100 RPM and 25 °C for 24 h. Following this, the suspension was vacuum filtered using Qualy filter paper (No.9) in a Buchner funnel connected to a vacuum pump (121, Primastec, Itú, Brazil). The filtrate was then concentrated using a rotary evaporator (Q344B2, Quimis, Diadema, Brazil) at 40 °C. Finally, the resulting solution was collected, its volume was determined using a graduated cylinder, and it was stored at 5 °C until the assays. The crude extract was subsequently diluted with water to achieve final concentrations of 0.1, 0.5, and 1 mg·mL^−1^.

### 2.3. Chemical Characterization of MEs

For Ultra-Performance Liquid Chromatography (UPLC) analysis, stock solutions (4 mg·mL^−1^) of the hydroalcoholic extracts from No, Pt, So, and Ts were prepared and subsequently diluted to a final concentration of 800 µg·mL^−1^ using an LC-MS-grade acetonitrile/methanol (1:1) mixture. Prior to analysis, all extracts were filtered through a 0.22 µm hydrophilic PTFE membrane (Filtrilo, Colombo, Brazil).

A 2 µL aliquot of each sample was injected into a UPLC Acquity system (Waters Co., Milford, MA, USA) coupled to a Xevo G2S Q-Tof mass spectrometer (Waters Co., Milford, MA, USA), which was equipped with an electrospray ionization (ESI) source, a quadrupole, and a time-of-flight (QTof) analyzer. Chromatographic separations were performed using a Hypersil GOLD column (50 × 2.1 mm i.d., 1.9 µm particle size, Thermo Scientific, Waltham, MA, USA), maintained at 40 °C, with a mobile phase flow rate of 0.3 mL.min^−1^. The mobile phases consisted of ultrapure water with 0.1% formic acid (pH 3.0) (mobile phase A) and LC-MS-grade acetonitrile (mobile phase B), following the gradient program: 0 min—90% A, 10% B; 0–1 min—90% A, 10% B; 1–3 min—70% A, 30% B; 3–8 min—70% A, 30% B; 8–12 min—10% A, 90% B; 14–15 min—90% A, 10% B; and 15–20 min for column equilibration.

Data acquisition was conducted in fast data-dependent acquisition (Fast DDA) and MSE (DIA) modes, utilizing argon as the collision gas and an energy range of 20–40 eV. Spectra were recorded in centroid mode for both positive and negative ionization modes within an *m*/*z* range of 50–1500, with a scan time of 0.1 s over a 20 min runtime. Each FDDA cycle included an m/z value corresponding to MS^1^ above a threshold of 20,000, where peaks exceeding this threshold were selected as precursor ions for fragmentation. MS/MS spectra were acquired while the signal remained above a threshold of 10,000. Nitrogen was used as the nebulizer gas, with a cone gas flow rate of 100 L·h^−1^ and a desolvation gas flow rate of 800 L·h^−1^. The sampling cone voltage and source offset were set at 40 V and 80 V, respectively. Accurate mass measurements were ensured using a lock spray reference solution containing leucine enkephalin (Leu-Enk, *m*/*z* 554.2615, [M−H]⁻; *m*/*z* 556.2771, [M+H]⁺). Desolvation and ionization temperatures were maintained at 350 °C and 120 °C, respectively.

For metabolite annotation, raw UHPLC-MS data (.D files) obtained in FDDA mode were directly uploaded and processed using MS-DIAL software (ver. 4.7). Data collection was performed with an MS^1^ tolerance of 0.02 Da and an MS^2^ tolerance of 0.06 Da. The molecular formula finder was restricted to the elements C, H, O, P, and N. Peak detection was configured with a minimum peak height amplitude of 2000 and a mass slice width of 0.1 Da. Deconvolution parameters were set as follows: a sigma window value of 0.1 and an MS/MS abundance cutoff of 10 amplitudes.

Metabolite annotation was conducted using the MS-DIAL metabolomics MSP spectral kit, which incorporates EI-MS, MS/MS, and CCS values for ESI (+ and −)-MS/MS data from both experimental and in silico databases (16,995 unique compounds and 15,245 unique compounds) [19]. Additional annotation was performed using MS-FINDER (for molecular formula prediction based on isotopic patterns and in silico fragmentation), SIRIUS (for molecular formula and structure prediction using isotopic patterns and fragmentation trees), and relevant literature sources. Candidate metabolites were identified by querying spectral databases, including MassBank, GNPS, KNApSAcK, PubCME, COCONUT, UNPD, NANPDB, NPA, PlantCyc, FooDB, T3DB, ChEBI, STOFF, LipidMAPS, YMDB, and ECMDB. Candidates were ranked based on similarity scores, which were computed by comparing experimental MS/MS spectra against database entries [19,20].

The top-ranked candidates were assigned, but in cases where key fragment ions were not adequately explained or similarity scores were identical, manual curation and cross-referencing with available literature were performed to refine metabolite identification [21].

### 2.4. Assessment of Conidia Germination and Mycelial Growth

Drops (10 µL) of a suspension containing 4 × 10⁴ conidia·mL^−1^ of *C. lindemuthianum* were pipetted onto polyethylene (Con-Tact, Plavitec, São Paulo, Brazil) slides, followed by the addition of 10 µL of sterile distilled water (control) or MEs (0.1, 0.5 or 1.0 mg·mL^−1^). The slides were kept in a humid chamber (100% RH) and incubated at 21 °C under a 12 h photoperiod. After 72 h, the percentage of conidia germination was evaluated under a light microscope at 400× magnification after adding a 10 µL drop of Amann’s blue dye. A conidium was deemed germinated if it developed a germ tube, regardless of its length. In each drop, 100 conidia were counted and classified as germinated or non-germinated [22]. For statistical purposes, each drop was considered a replicate.

To evaluate the effect of MEs on the growth of *C. lindemuthianum* mycelium, 10 mL of MEs solution was added to 15 mL of potato dextrose agar (PDA) medium at 70 °C, resulting in a final volume of 25 mL at 0.1, 0.5 and 1.0 mg·mL^−1^ [23]. A volume of 5 mL of this compound medium was added to each Petri dish. After solidification, each plate received an 8 mm diameter disk of the fungal culture. Then, plates were incubated at 21 ± 2 °C under a 12 h photoperiod for 15 days. Control dishes contained 15 mL of PDA medium supplemented with 10 mL of sterile distilled water. Mycelial growth was assessed by measuring colony diameter along two perpendicular axes using a digital caliper (Mitutoyo, Suzano, Brazil). Each Petri dish served as an experimental unit for mycelial growth assays.

### 2.5. Evaluation of MEs in the Severity of Anthracnose and Plant Grownth

#### 2.5.1. Treatments with MEs

Plants were treated by drenching on the day of and 3, 6, and 9 days after sowing with 3 mL MEs at 0.1, 0.5, or 1.0 mg·mL^−1^. Sterile distilled water (DW) served as control. Thereafter, one set of plants was used for infection assays and other for plant growth evaluation.

#### 2.5.2. Inoculation and Assessment of Anthracnose Severity

Eleven days after sowing, a third set of bean plants was simultaneously inoculated with a homogeneous suspension of *C. lindemuthianum* at 1 × 10^6^ conidia·mL^−1^ and incubated in high humidity conditions (25 °C, 99% relative humidity) for 48 h. Following the incubation period, plants were transferred back to the growth chamber for disease evaluation. The severity of anthracnose symptoms was assessed at 7 and 15 days after inoculation, with the aid of the scale proposed by Rava et al. [24], varying from 1 (no symptoms) to 9 (most plants dead).

#### 2.5.3. Measurement of Length of Plant Shoot and Roots

Eleven days after sowing, a set of bean plants was carefully taken out of the tubes, and their roots were thoroughly rinsed under running water. Then, the shoot length, root system length, and total plant height were measured using a plastic ruler.

#### 2.5.4. Determination of Fresh and Dry Weight

Shoots and roots were separated at the collar region with the aid of a scalpel. Then, both parts were weighed individually to determine their fresh weight. The dry weight was determined after drying seedlings at 65 °C for 72 h.

#### 2.5.5. Quantification of Leaf Pigments

Leaf pigments were quantified as previously described [25], with modifications. For that, at eleven days after sowing, 8–10 disks (8 mm in diameter; about 100 mg) were taken from equidistant points on the primary leaf blade of a second set of plants, placed in Falcon tubes containing dimethyl sulfoxide (DMSO; 7 mL) and heated at 65 °C for 2 h. After this period, the volume in the tubes was completed to 10 mL with DMSO, and 250 µL aliquots of each sample was transferred to microplate wells. Absorbance was measured using a microplate reader (SpectraMax Paradigm, Molecular Devices, San Jose, CA, USA) at wavelengths of 480, 649, and 665 nm. Pigment concentrations were determined by the formulas described in Wellburn [26].

### 2.6. Experimental Design and Statistical Analysis

The experiments followed a completely randomized design with four replications. Each replication consisted of a Petri dish, a drop on a polyethylene film, and three tubes containing one plant each. These were used, respectively, to evaluate the effect of MEs on mycelial growth in solid medium, *C. lindemuthianum* conidial germination, and in vivo assays assessing anthracnose severity and early plant development.

After verifying homogeneity of variances, datasets were subjected to analysis of variance (ANOVA) followed by Tukey’s test at 5% significance level for mean separation or regression analysis (foliar pigments). Statistical analyses were carried out in R environment, version 4.3.0.

## 3. Results

### 3.1. Chemical Profiling of MEs

UPLC analysis of *N. oculata, P. tricornutum*, *T. obliquus,* and *T. tetrathele* extracts identified 32 compounds across one or more extracts, each belonging to distinct chemical and functional structural groups. This highlights the complexity and heterogeneity of the chemical composition of the extracts (Table 2). The compounds identified include monosaccharides, disaccharides, specific compound derivatives, amino acids and betaines, organic acids, terpenoids and their derivatives, lipids and their derivatives, polyphenols, and antioxidants.

Lipids, which constituted 34% of the 32 identified compounds, were the most prevalent and specific class, with 64% of these compounds being unique to a single microalgae extract. In comparison, 38% of the compounds were common to all four microalgae extracts, including amino acids and betaines, monosaccharides, disaccharides, terpenoids, and organic acids such as linoleic acid. Of the detected compounds, 28% were present in only one extract, 25% in two extracts, and 9% in three extracts.

Regarding the different microalgae species and their respective extracts, it was observed that the *P. tricornutum* extract contained the highest number of characterized compounds, accounting for 75% of the total identified. The *N. oculata* extract followed, containing 62% of the compounds, while the *T. obliquus* and *T. tetrathele* extracts each contained 59% of the compounds (Table 2).

### 3.2. Effect of MEs on the Conidial Germination and Mycelial Growth of C. lindemuthianum

**Figure 1:**
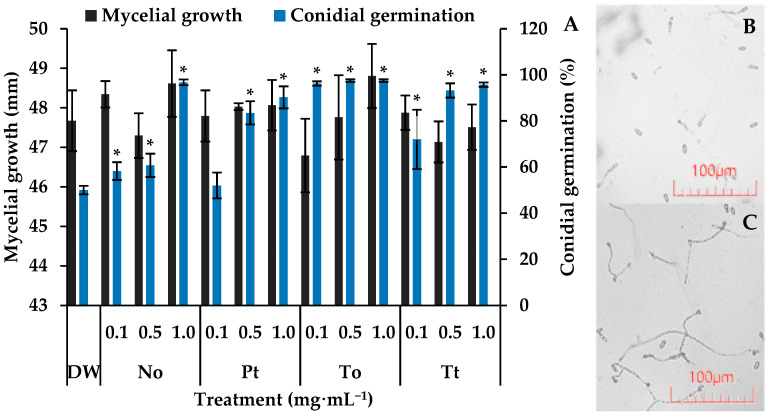
Mycelial growth (mm) and germination of conidia of *Colletotrichum lindemuthianum* incubated for 7 days and 72 h, respectively, in distilled water (DW) and hydroalcoholic extracts of *Nannochloropsis oculata* (No), *Phaeodactylum tricornutum* (Pt), *Tetradesmus obliquus* (To), or *Tetraselmis tetrathele* (Tt) at 0.1, 0.5 or 1.0 mg·mL^−1^ (**A**). Micrograph of conidial germination in DW (**B**) and To at 1.0 mg·mL^−1^ after 72 h of incubation (**C**). * indicate significant differences in relation to the control (DW) (Tukey test, *p* ≤ 0.05, n = 4). Error bars represent the standard deviation of the means.

On control slides, the percentage of germination of *C. lindemuthianum* conidia was 50%. In this situation, all of the tested MEs increased significantly spore germination by 80%, 92%, 94%, and 94% at 1.0 mg·mL^−1^ for *P. tricornutum*, *T. tetrathele*, *N. oculata*, or *T. obliquus*, respectively (Figure 1).

After seven days of incubation, C. lindemuthianum colonies in the control Petri dishes reached an average diameter of approximately 47.7 mm (Figure 1). Under these conditions, none of the four microalgae species at different concentrations affected the fugal growth, which remained around 47.8 mm.

### 3.3. Effect of MEs of Anthracnose Severity

The scores of anthracnose evaluated at seven and twelve days after inoculation of control bean plants were about seven and eight, respectively (Table 3). In this situation, no effect on the anthracnose severity was detected when applying the tested MEs.

### 3.4. Effect of MEs on Bean Plant Length of Plant Shoot, Roots, and Total

**Figure 2:**
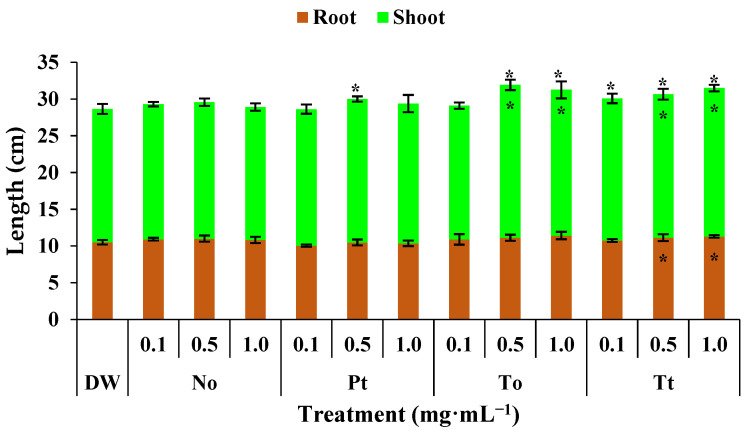
Total, root, and shoot lengths (cm) of bean plants (*Phaseolus vulgaris*) drenched four times with varying concentrations of hydroalcoholic extracts from *Nannochloropsis oculata* (No), *Phaeodactylum tricornutum* (Pt), *Tetradesmus obliquus* (So), or *Tetraselmis tetrathele* (Tt). Plants were grown at 21 ± 2 °C, with a photoperiod of 12 h and relative humidity of 80%, for 11 days after sowing. * Within the columns indicate significant differences between treatments and the control for root or shoot length, while outside the columns indicate differences for total length (root + shoot) according to Tukey’s test (*p* ≤ 0.05, n = 4). Error bars represent standard deviation of means.

In control bean plants, the root, shoot, and total plant lengths were 10.5 cm, 18.1 cm, and 28.6 cm, respectively (Figure 2). Under these conditions, hydroalcoholic extract of *P. tricornutum* increased the total length of plants by 4% at 0.5 mg·mL^−1^. The extract of *T. obliquus* increased the length of shoots and entire bean plants by 11% at both 0.5 and 1.0 mg·mL^−1^. Finally, the extract of *T. tetrathele* increased the length of entire bean plants by 7% at all tested concentrations and the length of shoots and roots by 6%.

### 3.5. Effect of MEs on Fresh and Dry Weight Plant

**Figure 3:**
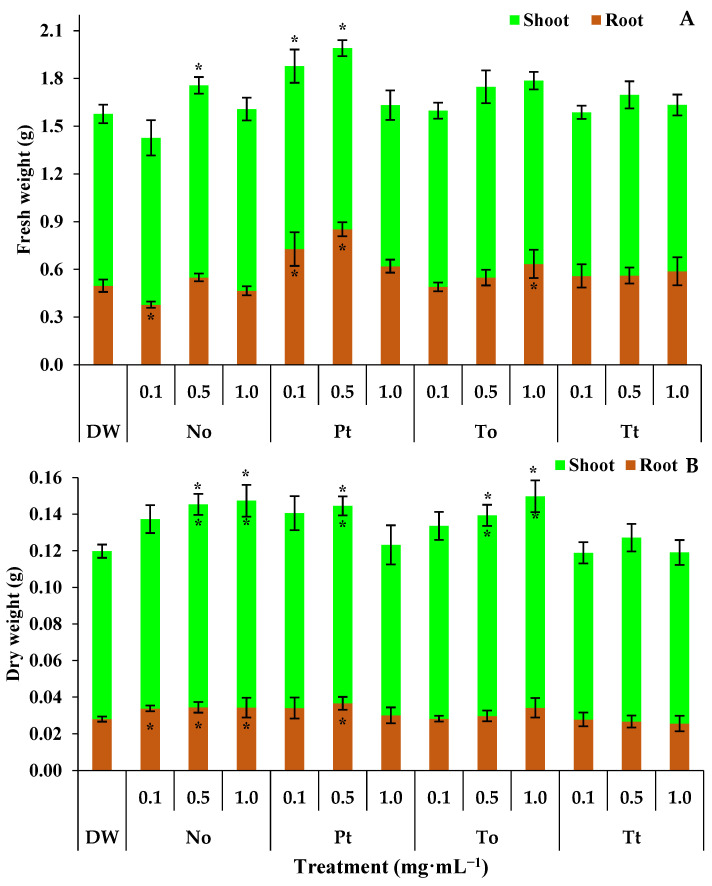
Root, shoot, and total fresh weight (**A**) and dry weight (**B**) (g) of bean plants (*Phaseolus vulgaris*) drenched four times with varying concentrations of hydroalcoholic extracts *Nannochloropsis oculata* (No), *Phaeodactylum tricornutum* (Pt), *Tetradesmus obliquus* (To), or *Tetraselmis tetrathele* (Tt). The plants were cultivated in a growth chamber under controlled conditions: 21 ± 2 °C temperature, 12 h photoperiod, and 80% relative humidity, for 11 days post sowing. * Within the columns indicate significant differences between treatments and the control for root or shoot fresh/dry weight, while outside the columns indicate differences for total fresh/dry (root + shoot) according to Tukey Test (*p* ≤ 0.05, n = 4). Error bars represent standard deviation of means.

The fresh weight of the root, aerial part, and entire bean plants treated with distilled water (DW) were 0.50 g, 1.08 g, and 1.58 g, respectively (Figure 3A). In these circumstances, the ME of *N. oculata* at 0.1 mg·mL^−1^ reduced the root fresh weight by 24%. On the other hand, at 0.5 mg·mL^−1^, the ME of *N. oculata* increased the fresh weight of entire bean plants by 11% (Figure 3A). The ME of *P. tricornutum* at 0.1 and 0.5 mg·mL^−1^ increased the fresh weight of roots by 46% and 70%, respectively, and entire bean plants by 19% and 25%, respectively. Finally, the ME of *T. obliquus* increased the fresh weight of roots by 26% at 1.0 mg·mL^−1^ (Figure 3A).

The dry weights of the shoots, roots, and whole bean plants in the water-treated control were 0.0918 g, 0.0280 g, and 0.1198 g, respectively (Figure 3B). Under these conditions, the ME of *N. oculata* increased the dry weight of roots by 22% at 0.1, 0.5, and 1.0 mg·mL^−1^ (Figure 3B). Also, the ME of *N. oculata* increased the dry weights of shoots and whole bean plants by 22% at both 0.5 and 1.0 mg·mL^−1^. On the other hand, the ME of *P. tricornutum* increased the dry weights of shoots, roots, and whole bean plants by 22% only at 0.5 mg·mL^−1^ (Figure 3B). Finally, the ME of *T. obliquus* increased the dry weights of shoots and whole bean plants by 22% at both 0.5 and 1.0 mg·mL^−1^.

### 3.6. Effect of MEs on Leaf Pigments

**Figure 4:**
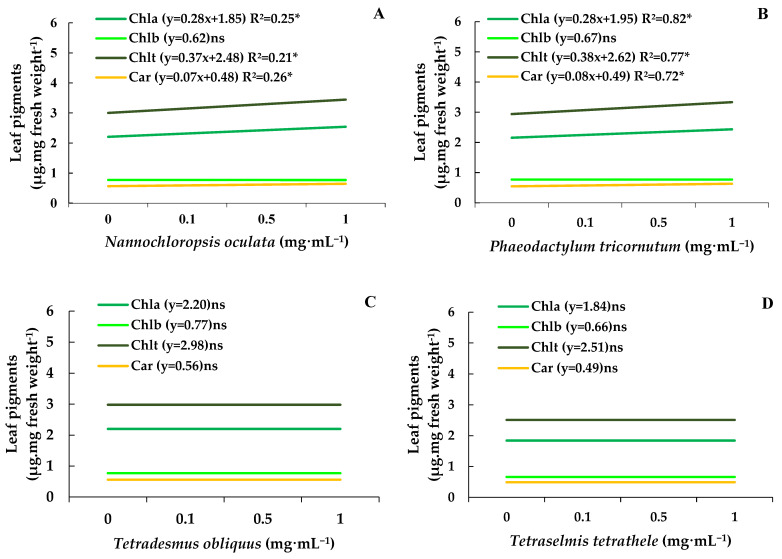
Chlorophylls a and b content, total chlorophyll, and carotenoid levels (µg·mg of fresh mass^−1^) in leaves of bean plants (*Phaseolus vulgaris*) drenched with varying concentrations of the microalgae *Nannochloropsis oculata* (**A**), *Phaeodactylum tricornutum* (**B**), *Tetradesmus obliquus* (**C**)*,* or *Tetraselmis tetrathele* (**D**). Plants were kept in a growth chamber at 21 ± 2 °C, with a 12 h photoperiod and 80% relative humidity, for 11 days after sowing. Equations marked with * indicate significant regression, while ‘ns’ means non-significant based on the F test (*p* ≤ 0.05; n = 4).

Chlorophylls a, b, and total and carotenoids determined on control bean plants were about 2.11, 0.74, 2.86, and 0.54 µg·mg of fresh weight^−1^ (Figure 4A–D). Under these circumstances, the MEs of *N. oculata* and *P. tricornutum* at 1.0 mg·mL^−1^ increased the chlorophyll and total chlorophyll and carotenoids levels by 15%, without affecting the content of chlorophyll b (Figure 4A,B). In contrast, the MEs from *T. obliquus* and *T. tetrathele* had no impact on the leaf pigment content (Figure 4C,D).

## 4. Discussion

The continuous need to increase productivity without expanding the agricultural frontiers together with the growing concerns regarding pesticide residues in both the environment and in food have driven the search for sustainable productions systems. In this plot, the use of microalgae arises as an eco-friendly strategy for both increasing yield and protecting plants against biotic and abiotic factors [27,28,29].

Most of the identified compounds present in MEs tested in the present study belong to the lipids chemical group. Indeed, microalgae can accumulate higher amounts of lipids (up to 80% of dry weight) when compared to macroalgae (up to 5%). Among accumulated lipids, fatty acids are the most common in microalgae content [30]. Curiously, the biostimulant effect observed in wheat plantlets after treatment with an *Arthrospira platensis* extract has been attributed to fatty acids [30].

Amino acids and betaines were also found in MEs tested in our work. Among several compounds listed in these groups, glycine betaine, a small organic molecule, can play a vital role in helping plants cope with various environmental stresses. Apparently, glycine betaine acts as an osmolyte, a molecule that helps plants maintain cell turgor (pressure) by balancing the concentration of solutes within the cell [31]. Amino acids, on the other hand, could be precursors of key phytohormones such as auxin and salicylic acid and other biomolecules including polyamines and phenolic compounds [27,28].

Sugars and their building blocks were also found in MEs used in our research. These molecules are well known inducers of plant growth and protection against both biotic and abiotic stresses [27,28]. Thus, for instance, polysaccharides extracted from *P. tricornutum*, *Scenedesmus* sp. and *Porphyridium* sp. increased the activity of defense-related enzymes such as phenylalanine ammonia liase, chitinase, β-1,3-glucanase, and peroxidases in tomato plants [32]. Monosaccharides and disaccharides present in microalgae’s biomass can act as biostimulants by providing energy and modulating hormonal responses [33,34], regulating osmotic balance [35,36], promoting microbial activity, and stimulating root growth [37,38].

Organic acids, terpenoids, polyphenols, and antioxidants were present in the tested MEs. Biomolecules belonging to these groups are known for possessing a wide range of biological activities [39,40]. For instance, phloroglucinol and its derivatives have potential antibacterial, antifungal, and antiviral properties [40]. Also, phloroglucinol and eckol seem to play a significant role in activating biochemical pathways essential for enhancing crop productivity [39]. Resveratrol, another polyphenol, can stimulate the growth of lettuce, probably by reducing the production of reactive oxygen species and by increasing the photosynthetic efficiency [41]. Given that citric acid can enhance plant growth and stress tolerance through improved photosynthesis, antioxidant defenses, and heavy metal chelation, it is reasonable to suppose that citric acid produced by microalgae and applied to plants can have similarly positive effects [42]. In fact, several studies have confirmed that exogenous citric acid application enhances growth, yield, and photosynthetic pigments in *Gossypium barbadense* (cotton) [43], boosts germination rates in *Carica papaya* (papaya) [44], increases growth and chlorophyll content in *Phaseolus vulgaris* (common bean) [43], and enhances photosynthetic pigments in *Zea mays* (maize) [45].

In the present work, all tested MEs increased the in vitro germination of *C. lindemuthianum* conidia. These results could be explained by the presence of sugars in the MEs. It is well known that sugar monomers can stimulate the growth of fungi including *Colletotrichum* [46]. Therefore, it would be expected that the presence of some sugars in MEs could accelerate the germination and growth of *C. lindemuthianum.*

The mycelial growth of *C. lindemuthianum* was not affected by any of the tested microalgae. In contrast, it has been demonstrated that aqueous extracts of *Nannochloropsis* sp., *Phaeodactylum* sp. and *Scenedesmus* sp. can reduce the mycelial growth of the plant pathogenic fungi *Sclerotium rolfsii, Rhizoctonia solani,* and *Botrytis cinera* [47]. These differences may be attributed to variations in methodology between our study and previous research. While Schmid et al. [47] spread the extracts over the semi solid PDA medium, we added them into it. Therefore, in the first, the fungus would be directly in contact with the extracts. Alternatively, our results could be explained by a different degree of sensibility of *C. lindemuthianum* to extracts of the tested microalgae species. For instance, the aqueous extract of *S. obliquus* both reduces and increases the mycelial growth of *B. cinerea* and *Alternaria alternata,* respectively [47].

None of the tested microalgae affected the anthracnose severity in bean plants. Interestingly, it has been reported that microalgae extracts can activate a wide range of defenses responses and protect plants against pathogens. For instance, the spraying of *Chlorella fusca* cells reduced the severity of *Colletotrichum orbiculare* in cucumber [48]. Two hypotheses can be raised to explain these disparities. Firstly, this could be attributed to the material applied to the leaves, while we sprayed a hydroalcoholic extract, Kim et al., [48] used live *C. fusca* cells probably with the growth medium. Therefore, some exopolysaccharides released by *C. fusca* in the growth medium would be present and could have induced defense responses in cucumber leaves. Alternatively, it is known that the conditions used during the production can directly affect the composition of microalgae [27]. Consequently, the biological effect could be entirely different. Therefore, evaluating the biological activity of microalgae grown under different conditions will be an exciting opportunity for future studies.

Extracts from tested microalgae species variably enhanced certain growth variables of bean plants, including root and shoot length, as well as their fresh and dry weights. Indeed, studies have shown that microalgae can promote the growth of various plant species, including *Beta vulgaris, Solanum lycopersicum, Triticum aestivum,* and *Phaseolus vulgaris* [30,32,49]. Despite the fact that the exact mode of action in plants is still unclear, it has been proposed that the biostimulant activity of microalgae is associated with the presence of pytohormones such as auxins, gibberelins and cytokinins, sugars, proteins, and aminoacids and antioxidant biomolecules [27,28]. These molecules could act alone or, more probably, in synergy.

The MEs of *Nannochloropsis oculata* and *Phaeodactylum tricornutum* significantly increased chlorophyll, total chlorophyll, and carotenoid levels in bean plants. Chlorophyll retention is a well-documented plant response to abiotic stress. For instance, exposure to specific chemicals, such as ethylene inhibitors, can delay senescence and chlorophyll degradation [50,51]. Interestingly, only these two microalgae species demonstrated this effect; however, no correlation with their chemical profiles was found, leaving the underlying mechanism unresolved.

## 5. Conclusions

In sum, our results show that hydroalcoholic extracts of the microalgae *P. tricornutum*, *N. oculata*, *T. tetrathele*, and *T. obliquus* are composed by myriad of biomolecules including lipids, sugars, aminoacids, organic acids, terpenoids, polyphenols, and antioxidants. Additionally, the tested MEs can biostimulate the initial growth of bean plants without affecting the severity of anthracnose caused by *C. lindemuthianum*. The microalgae tested in our work present potential applications in sustainable agriculture as natural plant growth enhancers. These findings support the exploration of microalgal extracts as eco-friendly alternatives to synthetic agrochemicals, contributing to more sustainable crop production practices.

## Figures and Tables

**Table 1:** Species, strain, source and abbreviation of the microalgae used in the study.

Species	Strain	Source	Abbreviation
*Nannochloropsis oculata*	CCMP525	Bigelow National Center for Marine Algae and Microbiota (NCMA), Boothbay, ME, USA	No
*Tetraselmis tetrathele*	-	Phytoplankton and Marine Microorganisms Ecology Laboratory at the Federal University of Rio Grande, Rio Grande, Brazil	Tt
*Phaeodactylum tricornutum*	CCAP 1055/1	Culture Collection of Algae and Protozoa, Scotland, UK	Pt
*Tetradesmus obliquus*	LCA-01	Laboratory of Algae Cultivation, Federal University of Santa Catarina, Florianopolis, Brazil	To

**Table 2:** Metabolic profile by UPLC of extracts of the microalgae *Nannochloropsis oculata* (No), *Phaeodactylum tricornutum* (Pt), *Tetradesmus obliquus* (To), and *Tetraselmis tetrathele* (Tt).

Compost	Average Rt (min)	Average *m*/*z*	Post Curation Result	Formula	MS-FINDER Score	MS-DIAL Score	No	Pt	To	Tt
1	0.524	217.0483	Phloroglucinol derivative	C_6_H_14_O_6_	-	-	976	21	12	2709
2	0.526	233.0643	Sedoheptulose	C_7_H_14_O_7_	-	94.4	79	7109	109	71
3	0.526	279.0569	2,5-Dimethyl-4-hydroxy-3(2H)-furanone	C_6_H_8_O_3_	-	-	31	2418	58	59
4	0.526	209.0663	Monossacharide	C_7_H_14_O_7_	-	100	7	786	5	5
5	0.527	365.1056	Dissacharide I	C_12_H_22_O_11_	-	84.3	508	494	8981	1495
6	0.535	236.1499	Glyceryl-trimethylhomoserine	C_10_H_21_NO_5_	-	82.9	5055	310	2914	6285
7	0.538	205.069	Galactitol	C_6_H_14_O_6_	8.2	-	7301	136	182	7212
8	0.55	118.0871	Glycine betaine	C_5_H_11_NO_2_	-	85.8	7058	68,501	141	7614
9	0.551	138.0554	2-methyl-3-vinyl maleimide	C_7_H_7_NO_2_	6.57	81.3	50,750	3385	54	75,166
10	0.556	251.061	Resveratrol	C_14_H_12_O_3_	-	99.9	909	70	70	2284
11	0.559	191.0192	Citric acid	C_6_H_8_O_7_	5.3	100	56	2750	294	60
12	0.56	160.1334	Aminovaleric acid betaine	C_8_H_17_NO_2_	-	64.5	4452	42	26	4789
13	0.563	387.1142	Dissacharide II	C_12_H_22_O_11_	7.61	96.7	15	7	1726	36
14	3.318	217.1078	(4S,5S,6E,8S,10R)-4,5,8-Trihydroxy-10-methyl-3,4,5,8,9,10-hexahydro-2H-oxecin-2-one	C_10_H_16_O_5_	-	99.9	7052	9065	3375	6587
15	11.267	415.2118	Terpenoid II	C_24_H_30_O_6_	-	100	6163	6448	5102	5210
16	11.479	325.2377	allo-Protolichesterinic acid	C_19_H_32_O_4_	5.07	100	119	4336	165	110
17	11.479	509.2704	MGMG 16:3	C_25_H_42_O_9_	-	100	12	3680	58	17
18	11.653	302.3056	Sphinganine	C_18_H_39_NO_2_	8.02	88.3	25,145	24,398	24,353	4745
19	11.714	675.358	DGMG 18:4	C_33_H_54_O_14_	-	-	8	2306	30	57
20	11.944	520.3621	LysoDGTSA 20:5	C_30_H_49_NO_6_	-	-	172	2123	31	251
21	12.003	377.2681	Diterpenoid	C_23_H_36_O_4_	-	-	23	2809	41	37
22	12.189	546.379	LysoDGTS 22:6	C_32_H_51_NO_6_	-	-	26	3354	34	28
23	12.225	353.1991	Unkown	C_19_H_30_O_6_	-	99.4	700	287	236	210
24	12.232	555.2836	SQMG(16:0/0:0)	C_25_H_48_O_11_S	7	-	11	578	6	31
25	12.238	374.3637	21-aminodocosane-2,3,20-triol (aminolipid)	C_22_H_47_NO_3_	-	86.7	4625	3953	4271	845
26	12.247	568.341	LysoPC 22:6	C_30_H_50_NO_7_P	-	-	14	2234	15	20
27	12.656	311.2012	Dictyochromenol	C_21_H_28_O_2_	6.76	86.1	3697	1662	272	3670
28	13.097	637.3025	Terpenoid II	C_36_H_44_O_10_	-	90.1	58	43	263	2334
29	13.294	815.494	SQDG (18:3/16:0)	C_43_H_76_O_12_S	-	-	6	27	1566	22
30	13.469	256.2647	Palmitamide	C_16_H_33_NO	-	null	117	93	2714	187
31	13.527	279.2317	Linoleic acid	C_18_H_32_O_2_	-	100	157	154	244	656
32	13.766	981.5798	DGDG (35:6)	C_52_H_86_O_17_	-	-	6	7	1178	55

**Table 3:** Anthracnose severity determined at 7 and 12 days after inoculation (Dai) of 11 days-old-bean plants (*Phaseolus vulgaris*) drenched four times with 3 mL of different concentrations of hydroalcoholic extracts from four microalgae species.

	Concentration (mg·mL^−1^)							
Microalgae Species	0.0	0.1	0.5	1.0	0.0	0.1	0.5	1.0
	7 Dai *				12 Dai			
*Nannochloropsis oculata*	6.3	5.8	6.4	6.1 ^ns^	8.0	7.7	8.2	8.1 ^ns^
*Phaeodactylum tricornutum*	8.2	7.2	7.3	7.0 ^ns^	8.5	8.1	8.4	8.1 ^ns^
*Tetradesmus obliquus*	7.2	6.6	6.4	6.4 ^ns^	8.0	8.0	8.1	8.0 ^ns^
*Tetraselmis tetrathele*	6.3	6.3	6.3	6.1 ^ns^	7.8	7.8	7.8	8.0 ^ns^

Plants inoculated with *Colletotrichum lindemuthianum* were kept in a humid chamber for 48 h and subsequently in a greenhouse at 25 ± 5 °C with a natural photoperiod and relative humidity of 80% for 12 days. To assess symptoms, the diagrammatic rating scale proposed by Rava et al. [24] was used as a basis. * dai: days after inoculation; ^ns^: not significant (ANOVA, *p* ≤ 0.05).

## Data Availability

The original contributions presented in this study are included in the article. Further inquiries can be directed to the corresponding author.
